# Chemical defense acquired via pharmacophagy can lead to protection from predation for conspecifics in a sawfly

**DOI:** 10.1098/rspb.2022.0176

**Published:** 2022-07-13

**Authors:** Pragya Singh, Neil Grone, Lisa Johanna Tewes, Caroline Müller

**Affiliations:** Chemical Ecology, Bielefeld University, Universitätsstr. 25, 33615 Bielefeld, Germany

**Keywords:** pharmacophagy, automimicry, plant–insect interaction, phytochemicals, *Hierodula patellifera* (Mantidae), Hymenoptera

## Abstract

Chemical defense is a widespread anti-predator strategy exhibited by organisms, with individuals either synthesizing or extrinsically acquiring defensive chemicals. In some species, such defences can also be transferred among conspecifics. Here, we tested the effects of pharmacophagy on the defense capability of the turnip sawfly, *Athalia rosae*, which can acquire *neo*-clerodane diterpenoids (clerodanoids) *via* pharmacophagy when having access to the plant *Ajuga reptans.* We show that clerodanoid access mediates protection against predation by mantids for the sawflies, both in a no-choice feeding assay and a microcosm setup. Even indirect access to clerodanoids, via nibbling on conspecifics that had access to the plant, resulted in protection against predation albeit to a lower degree than direct access. Furthermore, sawflies that had no direct access to clerodanoids were consumed less frequently by mantids when they were grouped with conspecifics that had direct access. Most, but not all, of such initially undefended sawflies could acquire clerodanoids from conspecifics that had direct access to the plant, although in low quantities. Together our results demonstrate that clerodanoids serve as a chemical defense that can also be transferred by interactions among conspecifics. Moreover, the presence of chemically defended individuals in a group can confer protection onto conspecifics that had no direct access to clerodanoids.

## 1. Introduction

Predation is an important interaction shaping communities and driving the evolution of species. Many individuals protect themselves against predation using chemical defenses that turn them distasteful, toxic and/or less nutritional [1,2]. Such defensive chemicals can either be synthesized *de-novo* or acquired extrinsically from their diet [3–5]. For example, the oleander aphid, *Aphis nerii*, sequesters cardenolides from its host plant during feeding and uses these defensive compounds against both vertebrate and invertebrate predators [6]. Alternatively, organisms can specifically take up defensive chemicals from plants *via* pharmacophagy [7–9]. For example, adults of some danaine butterfly species actively incorporate defensive chemicals like pyrrolizidine alkaloids from sources such as dried plant parts [10,11]. While these acquired chemicals confer protection on the individual taking them up, it is less well elucidated whether and how this protection can extend to conspecifics that may not have access to these chemicals directly from the source.

The possibility that chemically defended individuals confer protection from predation on initially undefended conspecifics can be realized *via* different means. Individuals may acquire such defensive chemicals directly from the (plant) source or indirectly via intraspecific [10,12] or interspecific [11,13] interactions. Alternatively, after attacking a chemically defended individual, a predator may not attack even chemically undefended conspecifics of the prey if it associates the phenotype with distastefulness by learned aversion or avoidance [14–16]. This phenomenon is often seen in combination with automimicry, wherein undefended individuals (mimics) benefit from the unpalatability of defended individuals (models) [17,18]. Furthermore, unpalatability or unprofitability of organisms can be associated with bright or aposematic coloration that functions as a warning signal to predators [19,20]. There is usually positive density-dependence in aposematism, such that conspicuous warning signals are more effective when they are common [20,21].

Understanding how the presence of chemically defended individuals affects the rest of the population is an important question as studies have shown that there can be intraspecific variation in chemical defense, with some individuals lacking chemical defenses entirely [22,23]. Such variation could arise if the defensive chemicals have an associated cost, for example, for acquisition and/or maintenance of the chemical defense [24]. Variation may also be influenced by intrinsic factors such as the age, sex, reproductive phase or immunological status of the individuals [25–27]. If defenses vary intraspecifically, the degree of protection from predation for chemically undefended individuals depends on the frequency of defended and non-defended individuals [28,29]. At a higher density of chemically defended individuals, the motivation of predators may be reduced to search for undefended prey [30], it may be harder to detect undefended prey [28,30], or more undefended individuals could indirectly access the chemicals and gain protection.

An excellent model system for examining the effect of defensive chemicals in deterring predation both directly and indirectly is the turnip sawfly, *Athalia rosae* (Hymenoptera: Tenthredinidae). The larvae of this species are well-studied for sequestering metabolites, i.e. glucosinolates, of their Brassicaceae host plants, which act as defense against various predators [31,32]. The bright orange adults still contain glucosinolates sequestered by the larvae [33], but do not seem to be protected by these compounds against predators such as birds and lizards [34,35]. However, *A. rosae* adults can additionally acquire other specialized metabolites, *neo*-clerodane diterpenoids (hereafter called ‘clerodanoids’), by pharmacophagy from certain plant species, such as *Ajuga reptans* or *Clerodendrum trichotomum* (both Lamiaceae) [36]. Some of the clerodanoids occurring in the host plant can be recovered unmodified in insect tissue, whereas others are likely slightly modified metabolic products from plant clerodanoid precursors [34,35]. Furthermore, clerodanoids can be acquired indirectly by nibbling on conspecifics that were exposed to plant material [12], without any visible damage to the conspecific. While effects of clerodanoids on mating behaviour have been shown previously [35,37], empirical evidence for other functions such as in defense against predation is scarce and indirect [38]. In the laboratory, *A. rosae* are usually maintained without *A. reptans* leaves, suggesting that clerodanoid access is not essential for the sawflies' survival [9,12,35]. Moreover, there is evidence for costs associated with clerodanoid uptake, as *A. rosae* adults with clerodanoid access exhibit a reduced lifespan [39]. Thus, most likely clerodanoid status of *A. rosae* varies in the wild.

Here, we investigated whether uptake of clerodanoids can function as defense against predation both for focal individuals of *A. rosae* that directly acquired these compounds after access to plants, and indirectly for other conspecifics that came into contact with the focal individuals. While unpalatability studies are often performed using birds, spiders, or lizards as predators, other ecologically relevant predators such as mantids are tested less frequently. We used the mantid *Hierodula patellifera* as potential predator. The mantid occurs as an ambush predator among others on *C. trichotomum* [40]*,* which is visited by *A. rosae* for pharmacophagy [37]. We observed the response of mantid predators in no-choice feeding assays to sawflies that either had access to clerodanoids (and potentially other plant compounds) directly from plants or indirectly from conspecifics or had no access (experiment 1). Next, we investigated survivorship of sawflies with or without clerodanoid access in both presence and absence of a predator (experiment 2). Lastly, we investigated if the presence of sawflies with clerodanoid access conferred protection from predation on conspecifics with no clerodanoid access and if this varied with their relative abundance (experiment 3). We also analysed the clerodanoid content of sawflies from different treatments (experiment 3). We predicted that both direct and indirect clerodanoid acquisition should lead to protection for the sawflies from predation by mantids. Furthermore, presence of chemically defended sawflies should confer protection from predation for conspecific sawflies, with protection increasing with the proportion of chemically defended individuals in the group. We also expect sawflies without clerodanoid access that were grouped with conspecifics with clerodanoid access to acquire clerodanoids.

## 2. Material and methods

### (a) Rearing conditions and experimental treatments

The individuals of *A. rosae* used in this experiment were taken from a laboratory stock population established using adults collected in the surroundings of Bielefeld, Germany, and supplemented annually with field-caught insects. The stock population was kept in mesh cages (60 × 60 × 60 cm) at a 16 h : 8 h light : dark cycle, at room temperature and approximately 60% relative humidity. Approximately 40 females and 30 males were put in a cage and provided with *Sinapis alba* (Brassicaceae) plants for oviposition. Emerging larvae were raised on *Brassica rapa* var. *pekinensis* (Brassicaceae) plants. Males and females were collected and separated within two days of pupal eclosion. Adults were kept in a climate chamber at 20°C (16 h : 8 h light : dark cycle, 70% relative humidity) before being used in experiments 1 and 2, and were kept in the laboratory under regular rearing conditions for experiment 3. Adults were fed ad libitum with a 2% (v/v) honey solution that was replenished every other day.

Adults were randomly assigned to one of three experimental treatments: no clerodanoid access (C−); direct clerodanoid access (C+); and indirect clerodanoid access (AC+). For the C+ treatment, adults got access to a leaf section (0.8 cm^2^) of *A. reptans* for 48 h, while for C− no *A. reptans* leaf was provided. For the AC+ treatment, adults got access to a C+ conspecific of the same sex for 48 h. Plants of *S. alba* were grown from seeds in a climate chamber (20°C, 16 h : 8 h light : dark, 70% r.h.), while *B. rapa* and *A. reptans* were grown from seeds in a greenhouse (≥20°C, 16 h : 8 h light : dark, 70% r.h.).

Twenty-three sixth instar individuals of *H. patellifera* (Mantidae) were purchased (www.interaquaristik.de) and reared in individual cages (20 cm × 20 cm × 20 cm) in a climatized room (approx. 20°C) on a diet of crickets. The mantids were not exposed to sawflies before experiment 1 and were starved 48 h prior to experiment 1. Each mantid was offered a cricket directly before experiments 2 and 3 to avoid starvation effects over the experimental days, and those individuals that did not consume the cricket were excluded.

### (b) Experiment 1: no-choice feeding assay to examine the effect of clerodanoid access on predator deterrence

**Figure 1.**
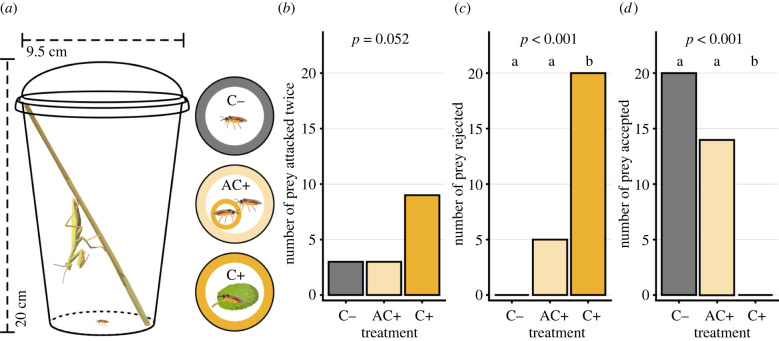
(*a*) Experimental design illustration of no-choice feeding assay, where each mantid was exposed to one *Athalia rosae* sawfly of different clerodanoid treatments (C−: no access, AC+: indirect access via conspecific that had contact with leaf of *Ajuga reptans*, C+: direct access to *A. reptans*) over multiple trials performed in different orders. Effects of clerodanoid treatment on number of prey (sawfly) that were (*b*) attacked twice (all other sawflies were attacked once), (*c*) rejected, and (*d*) accepted, by mantid (*n* = 20 replicates per treatment). Different letters denote significantly different (*p* < 0.05) treatment effects inferred from Tukey HSD post hoc tests in (*c*) and (*d*).

Mantids were placed individually in transparent containers (9.5 cm diameter, 20 cm height). One C−, C+ or AC+ female sawfly was introduced in the container (figure 1*a*) and the mantid**’**s response was recorded. Each assay was conducted at maximum for 15 min, but terminated when the sawfly was consumed earlier. The predator response variables examined during the assay were number of attacks on sawfly, prey rejection (see §1a,b in the electronic supplementary material), and prey acceptance (see §1c in the electronic supplementary material). Prey rejection was a distinct behaviour in which the mantid discarded the sawfly after mouth contact and could clearly be distinguished from the sawfly slipping or escaping from the mantid's grab. Prey acceptance was defined as the sawfly being consumed by the mantid. Note that even after initial prey rejection, the mantid could attack the sawfly again and consume it. Individuals that were attacked multiple times were recorded as positive for prey rejection if prey rejection behaviour was observed even once.

All assayed mantids were exposed to sawflies from the three experimental treatments, but in different orders. Two mantids did not attack sawflies from any of the treatments during the assay and were thus excluded from analysis as these mantids afterwards moulted. Six mantids received *A. rosae* in the order C+, AC+, C−, seven in the order C−, C+, AC+, and seven in the order AC+, C−, C+ (total number of replicates *N* = 20). Upon noticing the sawfly, the mantid would orient for an attack. However, we did not compare the latency until attack because the position of the mantids and how readily they noticed the sawflies differed between replicates. We did not examine the long-term survivorship of sawflies that were rejected by mantids, but damage to the sawfly spanned the spectrum from none to lethal (see electronic supplementary material §1a-c).

We expected mantids to attack C− sawflies but not AC+ or C+ sawflies, e.g. if there were any repellent olfactory cues associated with clerodanoid uptake by the sawflies. If AC+ or C+ individuals were attacked, we expected the mantids to reject the sawflies after tasting deterrent compounds and not to consume the prey.

### (c) Experiment 2: microcosm experiment to investigate clerodanoid access effect on survivorship in predator presence or absence

**Figure 2.**
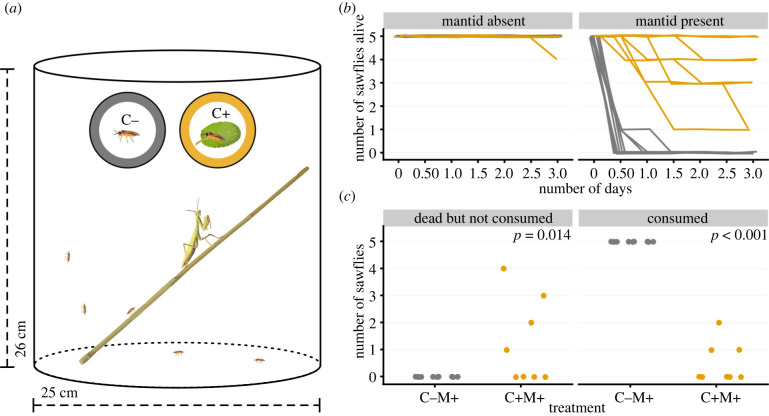
(*a*) Experimental design illustration of clerodanoid treatment (C−: no access, C+: access to leaf of *Ajuga reptans*) and predation microcosm experiment, where in each microcosm five *Athalia rosae* sawflies were added that were either C− or C+ and with or without a mantid. (*b*) Number of alive sawflies of different clerodanoid treatments over time. Lines are jittered to decrease overlapping. (*c*) Number of C− and C+ individuals that were ‘dead but not consumed’ or ‘consumed’ in replicates of mantid present (M+) treatment.

We used a fully factorial design (clerodanoid access × mantid presence) to evaluate the effect of clerodanoid acquisition on sawfly survival in the presence of a mantid predator in a microcosm (figure 2*a*). The four treatments were C− sawfly without mantid (C − M−), C− sawfly with mantid (C − M+), C+ sawfly without mantid (C + M−) and C+ sawfly with mantid (C + M+) with a sample size of eleven, ten, ten and eight, respectively. All trials were performed in microcosm cages (25 cm diameter, 26 cm height) with a honey-water supply. The cages were kept in a climate room at 20°C (16 h: 8 h light: dark cycle, 70% relative humidity) during the experiment. For each trial, we used five sawflies (three females and two males) and one or no mantid. We counted the number of sawflies alive every half-day for three days. For the mantid present trials, we also counted the number of sawflies ‘dead but not consumed’ at the end of three days, as sawflies may be attacked but not necessarily always completely consumed, e.g. if they are unpalatable. For these replicates, we calculated the number of consumed individuals as the difference between initial number of sawflies and the number of sawflies alive or ‘dead but not consumed’. This experiment was conducted two weeks after experiment 1. We expected C− with mantids to have reduced survival than other treatment sawflies. Moreover, we expected more C+ sawflies to be consumed by the mantids with time, which could be either due to a decreasing concentration of the clerodanoids or to a prolonged starvation of the mantids.

### (d) Experiment 3: predation on C− conspecifics in mixed groups of C+ and C− sawflies in microcosm

To test whether presence of C+ sawflies led to defense against predation also for C− sawflies, we set up four group-composition treatments each with six sawflies, consisting of varying relative abundance of C+ and C− individuals. From experiment 2 we knew that mantids can consume up to 5 C− *A. rosae* adults over a period of 1–3 half-days. The first group-composition treatment consisted of six C− sawflies (6C−), while the three mixed group-composition treatments were: two C+ and four C− sawflies (2C + 4C−), three C+ and three C− sawflies (3C + 3C−) and four C+ and two C− sawflies (4C + 2C−). For each replicate, we grouped the sawflies together in one Petri dish according to the assigned group-composition treatment 2 h prior to adding them to the microcosm to allow the sawflies to interact (e.g. mate or nibble).

We used 18 mantids for the experiment (the same mantids as used in previous experiments) and a microcosm cage set-up identical to experiment 2. Each mantid was exposed to all group-composition treatments in random order over multiple trials (four trials per mantid), with each trial lasting 2 days. Each mantid was fed a small cricket prior to each trial.

For the mixed group-composition treatments, we used only females as C− and males as C+ to distinguish between C− and C+ individuals in each replicate. Females can be identified by the black ovipositor visible on the orange abdomen. Both sexes can acquire clerodanoids [37] from a conspecific by nibbling, which is an aggressive interaction [35]. However, females are larger [41,42] and thus may nibble and acquire clerodanoids from C+ males more easily. Although we cannot rule out that mantids prefer male or female sawflies, we know that mantids consumed all C− sawflies irrespective of their sex (see §3b), while few C+ sawflies were consumed. At the end of each trial, we counted the number of alive or ‘dead but not consumed’ C+ and C− sawflies for each replicate. We calculated the number of consumed C− sawflies as the difference between initial number of C− sawflies and number of C− sawflies alive or ‘dead but not consumed’ at the end of the experiment. Similarly, we calculated the number of consumed C+ sawflies. We hypothesized that the presence and increasing abundance of C+ sawflies should lead to protection from predation for C− sawflies.

### (e) Chemical analysis of sawflies from experiment 3

To test whether C− sawflies in mixed group-composition treatments acquired clerodanoids compared to 6C− replicates, we collected C− sawflies from different group-composition treatments and analysed them chemically. We also collected C+ sawflies to confirm their clerodanoid acquisition. Lastly, we examined if C+ sawflies differed from C− sawflies of mixed group-composition treatments in their amounts of clerodanoids, i.e. if there is a difference between clerodanoid amounts acquired directly from leaves or indirectly from conspecifics. We only collected individuals from replicates that had intact sawflies remaining at the end of the trial and stored them at −80°C until further analysis. The final sample sizes for C− sawflies chemically analysed were six, five, seven, and five samples each of 6C−, 2C + 4C−, 3C + 3C− and 4C + 2C− group-composition treatments, respectively. The final sample sizes for C+ sawflies were one, four and five from 2C + 4C−, 3C + 3C− and 4C + 2C− group-composition treatments, respectively. Individuals were freeze-dried, homogenized and extracted twice in ethyl acetate (LC-MS grade, VWR, Leuven, Belgium). After centrifugation, the supernatants of the two extractions were pooled to a final volume of 400 µl for each individual. The extracts were dried in a vacuum centrifuge at 35°C, suspended in 125 µl 100% methanol (LC-MS grade, Fisher Scientific, Loughborough, UK) in an ultrasonic bath for 15 min, and filtered using syringe filters (polytetrafluoroethylene membrane, 0.2 µm pore size, Phenomenex, Torrance, CA, USA). The samples were analysed using an ultra high performance liquid chromatograph (UHPLC; Dionex UltiMate 3000, Thermo Fisher Scientific, San José, CA, USA) with a Kinetex XB-C18 column (1.7 µm, 150 × 2.1 mm, with guard column, Phenomenex), coupled to a quadrupole time of flight mass spectrometer (QTOF-MS; compact, Bruker Daltonics, Bremen, Germany), see electronic supplementary material §2 for details. The resulting chromatograms were processed with the software Compass Data Analysis 4.4 (Bruker Daltonics). The putative clerodanoids with neutral masses *m/z* of 482 (C_24_H_34_O_10_, called ‘clerodanoid 1’) and 484 (C_24_H_36_O_10_, called ‘clerodanoid 2’) occur in the chromatograms as [M + HCOOH-H]^−^ adducts resulting in features with *m/z* of 527.21 and 529.23, respectively [12]. These two yet unidentified clerodanoid candidates are considered as metabolic products of other clerodanoids from *A. reptans*, because they were not detectable in plant material [12]. We manually integrated the peak areas of these two features from the extracted ion chromatograms (*m/z* of 0.02 accuracy) as semi-quantitative proxy for the amounts present in the body. We expected C− sawflies from mixed group-composition treatments to acquire clerodanoids, but to have lower amounts of clerodanoids compared to C+ sawflies.

### (f) Statistical analyses

In experiment 1, we examined whether treatment had an effect on number of attacks, prey rejection, and prey acceptance using a binomial generalized linear mixed-effects model (GLMM), with mantid identity as random effect. As there was quasi-complete separation in our data for two variables (prey rejection and prey acceptance), i.e. the predictor variable could perfectly predict the response variable for a subset of our data, we fitted a Bayesian binomial GLMM using ‘blme’ package (v. 1.0-5) [43] for all variables. We evaluated whether the order in which the mantid was presented the treatments influenced the mantid response variables by incorporating the trial round of a treatment as a fixed effect (due to only three levels) in each model, and this gave qualitatively similar results with trial order having no significant effect (electronic supplementary material §3a).

In experiment 2, we examined the effect of treatment, time and their two-way interaction on number of alive sawflies using a Poisson GLMM (‘lme4’ package v. 1.1-27.1 [44]) with replicate ID as random effect. We also examined if there was a significant difference in number of consumed and ‘dead but not consumed’ sawflies between C− and C+ treatments with mantids (C−M+ and C+M+) using a Kruskal-Wallis test.

In experiment 3, we calculated the proportion of consumed, alive and ‘dead but not consumed’ C− and C+ sawflies with respect to the initial abundance of C− and C+ sawflies, respectively, in that replicate. We examined whether the proportion of consumed, alive and ‘dead but not consumed’ C− individuals differed significantly among the group-composition treatments using a binomial GLMM (‘lme4’ package), with mantid identity and trial number as random effects. Finally, we examined if there was a significant difference in the amount (semi-quantified based on peak area) of the two putative clerodanoids between C− and C+ sawflies of mixed group-composition treatments using a Kruskal-Wallis test. We also tested whether C− sawflies from mixed group-composition treatments had significantly different clerodanoid amounts from C− sawflies of the 6C− treatment using a Kruskal-Wallis test.

All data were analysed using R 4.0.5 (2021-03-31) [45]. We checked and tested model assumptions statistically and visually. *Post-hoc* tests were conducted using ‘multcomp’ package (v. 1.4-17) [46].

## 3. Results

### (a) Experiment 1. Clerodanoid access protects against consumption by predator

All sawflies, irrespective of treatment, were attacked at least once with no significant effect of treatment on number of attacks (*χ*^2^ = 5.92, d.f. = 2, *p* = 0.052; figure 1*b*). By contrast, prey rejection differed significantly between treatment (*χ*^2^ = 21.15, d.f. = 2, *p* < 0.001; figure 1*c*, electronic supplementary material §3b), with C+ individuals being rejected more often than C− (*post hoc* test: *p* < 0.001) and AC+ (*post hoc* test: *p* < 0.001). Likewise, treatment had a significant effect on whether an individual was consumed (*χ*^2^ = 21.28, d.f. = 2, *p* < 0.001; figure 1*d*, electronic supplementary material §3b), with C+ individuals (0% consumed) being significantly less consumed compared to C− (100% consumed) (*post hoc* test: *p* < 0.001) and AC+ (70% consumed) (*post hoc* test: *p* = 0.001). Note that C+ sawflies were usually attacked twice, as they were often initially rejected by mantids (figure 1*c*) but then re-attacked during the trial. By contrast, C− sawflies were never and AC+ sawflies were infrequently rejected by the mantids, and were consumed within the first attack (figure 1*d*).

### (b) Experiment 2. C+ individuals not consumed by predator even after prolonged exposure

There was a significant interactive effect of treatment and time on number of sawflies alive (*χ*^2^ = 36.76, d.f. = 3, *p* < 0.001). In the treatment with no mantids, all C− and all but one C+ individuals survived across all replicates (figure 2*b*). In C− treatments with mantids, no individual was alive after three half-days. By contrast, for C+ treatments with mantids, all individuals were alive in two replicates. In the other six C+M+ replicates the number of alive individuals decreased with time, although in no replicate were all individuals killed. The number of ‘dead but not consumed’ individuals significantly differed between C−M+ and C+M+ treatments (*χ*^2^ = 5.95, d.f. = 1, *p* = 0.014, figure 2*c*). Similarly, there was a significant difference in number of consumed sawflies (*χ*^2^ = 15.63, d.f. = 1, *p* < 0.001, figure 2*c*), with all sawflies consumed in the C−M+ treatment while only few sawflies were consumed across three replicates in the C+M+ treatment. In four C+M+ replicates but zero C−M+ replicates, we collected ‘dead but not consumed’ individuals, suggesting that while the mantids attacked C+ individuals, they were not always consumed.

### (c) Experiment 3. Few C− individuals consumed by predator if C+ individuals are present

Group-composition treatment had a significant effect on the proportion of consumed C− sawflies (*χ*^2^ = 41.87, d.f. = 3, *p* < 0.001; electronic supplementary material §4a), with significantly more C− consumed in the 6C- treatment compared to the other mixed group-composition treatments (table 1*a*). There was no significant difference between the three mixed group-composition treatments (table 1*a*). The proportion of alive C− sawflies differed between the treatments (*χ*^2^ = 27.23, d.f. = 3, *p* < 0.001; electronic supplementary material §4b), with significantly fewer C− alive in the 6C− compared to the 2C + 4C- and 3C + 3C− treatment, while other treatments were not significantly different (table 1*b*). Similarly, the proportion of ‘dead but not consumed’ C− sawflies differed between the treatments (*χ*^2^ = 8.77, d.f. = 3, *p* = 0.032; electronic supplementary material §4c), with significantly more C− sawflies being ‘dead but not consumed’ in the 4C + 2C− compared to the 6C− treatment, but no other significant differences between treatments (table 1*c*). Similar to experiment 2, C+ sawflies were consumed rarely, although in many replicates there were ‘dead but not consumed’ C+ individuals (electronic supplementary material §5a–c).

**Table 1.** Results of *post-hoc* analyses for dependent variables of experiment 3, where we examined the effects of different group-composition, i.e. varying relative abundance of *A. rosae* sawflies with (C+) and without (C−) access to a leaf of *A. reptans*, on proportion of (*a*) consumed, (*b*) alive, and (*c*) ‘dead but not consumed’ C− individuals. Significant differences (*p* < 0.05) are highlighted in bold.

pairwise comparison	(*a*) proportion of consumed C−				(*b*) proportion of alive C−				(*c*) proportion of ‘dead but not consumed’ C−			
	estimate	SE	*z* value	*p*(>/z/)	estimate	SE	*z* value	*p*(>/z/)	estimate	SE	*z* value	*p*(>/z/)
2C + 4C− versus 0C + 6C−	−2.14	0.51	−4.14	**<0**.**001**	1.67	0.50	3.29	**0**.**005**	0.94	0.46	2.01	0.182
3C + 3C− versus 0C + 6C−	−2.71	0.53	−5.06	**<0**.**001**	2.53	0.54	4.61	**<0**.**001**	0.73	0.51	1.41	0.487
4C + 2C− versus 0C + 6C−	−2.40	0.58	−4.12	**<0**.**001**	1.45	0.61	2.38	0.080	1.47	0.53	2.76	**0**.**029**
3C + 3C− versus 2C + 4C−	−0.57	0.49	−1.16	0.645	0.86	0.48	1.75	0.292	−0.20	0.49	−0.42	0.974
4C + 2C− versus 2C + 4C−	−0.26	0.57	−0.45	0.968	−0.21	0.57	−0.38	0.981	0.53	0.51	1.04	0.725
4C + 2C− versus 3C + 3C−	0.31	0.57	0.53	0.950	−1.07	0.60	−1.79	0.273	0.74	0.55	1.33	0.542

**Figure 3.**
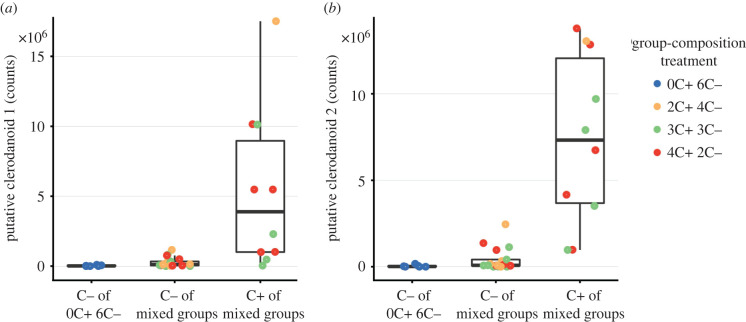
Amount (peak area) of chemical features representing putative clerodanoids in the body of *A. rosae* sawflies. (*a*) Clerodanoid 1 (C_24_H_34_O_10_) and (*b*) clerodanoid 2 (C_24_H_36_O_10_) were quantified from the extracted ion chromatograms for C− (without access to a leaf of *A. reptans*) and C+ (with access to a leaf of *A. reptans*) sawflies of different group-composition treatments. Mixed groups represent groups that had both C+ and C− sawflies present in the microcosm, while 0C + 6C− had only C− sawflies present.

The chemical analysis revealed that the clerodanoids 1 (482) and 2 (484) could be detected in 16 (approx. 94%) and 14 (approx. 82%), respectively, of the 17 sampled C− sawflies from mixed group-composition treatments (figure 3*a,b*), occurring as prominent peaks in the chromatograms (electronic supplementary material, figure 6). All ten (100%) C+ sawflies had acquired both clerodanoids (figure 3). There was intraspecific variation in the amount of clerodanoids acquired for both C+ and C− sawflies of mixed group-composition treatments. Two out of six replicates of the 6C− group-composition treatment also had small amounts of clerodanoids (figure 3), possibly resulting from contamination as these replicates were placed in the microcosms in which C+ individuals had previously been housed. This contamination may also explain why these C− sawflies were not consumed by the mantids. C+ sawflies had significantly higher amounts of both clerodanoids 1 (*χ*^2^ = 10.98, d.f. = 1, *p* < 0.001, figure 3*a*) and 2 (*χ*^2^ = 15.75, d.f. = 1, *p* < 0.001, figure 3*b*) than C− sawflies for mixed group-composition treatments. C− sawflies of mixed group-composition treatments had a significantly higher amount of clerodanoid 1 (*χ*^2^ = 5.02, d.f. = 1, *p* = 0.024, figure 3*a*) but not of clerodanoid 2 (*χ*^2^ = 3.77, d.f. = 1, *p* = 0.052, figure 3*b*) than C− of the 6C− treatment.

## 4. Discussion

Chemical defense as an anti-predator strategy is widespread and well-documented in animals [47]. An interesting aspect of such chemical defense is its transmission between conspecifics and how the presence of chemically defended individuals can confer protection onto undefended conspecifics. In some species, such defensive chemicals can be acquired pharmacophagously, as in the case of our study organism, *A. rosae*, which takes up and further metabolizes clerodanoids (and potentially also other chemicals) from *A. reptans* plants [9,37]. Here, we showed that prior access to *A. reptans* leaves provides a defense against predation by making the sawflies unpalatable to the predator. We also demonstrated that clerodanoid access provides protection not only to focal individuals but also to conspecifics in mixed groups of C+ and C− individuals.

While most sawflies without access to clerodanoids were lethally attacked and consumed by the mantids, only very few sawflies that had taken up clerodanoids from leaves were consumed, as we had predicted. Nevertheless, many sawflies with clerodanoid access were also attacked but then readily rejected by the mantids, as shown by the prey rejection behaviour in experiment 1 and by the higher number of ‘dead but not consumed’ sawflies in experiment 2. This rejection is likely induced by the bitter taste of the clerodanoids that may be deposited on the cuticle and in body tissue of the adult sawfly [38]. When tested directly, two other clerodanoids (clerodendrin B and D) had a deterrent effect on Japanese tree sparrows, which consumed fewer rice grains that had been treated with the clerodanoids compared to untreated grains [38]. A taste-rejection behaviour, in which predators taste but do not ingest a prey item, as found by the mantids in the present experiment, has been shown to be elicited by distasteful prey and can lead to an increased survivorship of the prey [48]. Although we did not quantify the long-term survivorship of sawflies after mantid attack in experiment 1, visual inspection showed that the damage spectrum ranged from nearly unharmed to dead sawflies (SI 1a,b), indicating that clerodanoid uptake could lead to survivorship advantages.

Adult *A. rosae* can acquire clerodanoids not only from plants but also from conspecifics *via* nibbling on their body surface. However, acquiring clerodanoids indirectly from conspecifics resulted in less protection than direct acquisition from the plants in *A. rosae* in our experiments. Not all sawflies successfully acquire detectable clerodanoid amounts from C+ conspecifics [12] and the concentrations are usually much lower than after direct uptake from the *A. reptans* leaves (figure 3). Such quantitative and potentially also qualitative differences in clerodanoid acquisition may explain why many AC+ sawflies (experiment 1) and C− sawflies in mixed group-composition treatments (experiment 3) were consumed by mantids. The effectiveness of defense chemicals sequestered from host plants against predators has been shown to be concentration-dependent in a glucosinolate-sequestering leaf beetle species, with individuals having lower levels of sequestered glucosinolates being more susceptible to predation [49].

In *A. rosae*, transfer of clerodanoids can occur both within and between sexes [12], which seems rather exceptional. In other insect species, usually chemicals are transferred from the male to the female during mating. For example, in some arctiid moth species, pyrrolizidine alkaloids are sexually transmitted from males to females, and these chemicals render protection against predation to the recipient female [50,51]. Moreover, such defensive chemicals acquired by the female from the male can also be incorporated into the offspring [52–54], providing it with benefits. Evidence for such benefits of parental clerodanoid provisioning to offspring in *A. rosae* is currently lacking [9].

Our data from the predation microcosm experiment (experiment 2) demonstrated that the mantids attacked C+ sawflies, with numbers of alive sawflies decreasing over time, but this decline was less rapid than that of C− sawflies in presence of mantids. This suggests that the mantids may learn to avoid C+ sawflies after first encounters, leading to a longer survival period of the sawflies. Learned aversion has been found in the mantid *Tenodera aridifolia*, which reduced attacks on prey items that were made bitter [55]. Similarly, repeated exposure to unpalatable milkweed bugs that had sequestered cardenolides led to avoidance by *T. aridifolia* of both palatable and unpalatable milkweed bugs altogether [14]. Interestingly, the mantid *H. patellifera* attacked sawflies in both experiments 2 and 3 despite having been exposed to the C+ sawflies previously, suggesting that such avoidance learning may not last for long, as also seen in *T. aridifolia* [56]. Moreover, avoidance learning can be more effective if the distasteful prey is conspicuously coloured [57,58]. Indeed, organisms that use chemical defenses are often brightly coloured and conspicuous, i.e. aposematic, to advertise their distastefulness [20,59]. The sawfly *A. rosae*, used in our study, is aposematically coloured, with a bright orange body (electronic supplementary material §1), which might facilitate temporary avoidance learning by mantids. It remains to be tested whether other species or even adults of *H. patellifera* are likewise deterred by clerodanoids. The effectiveness of chemical defenses can differ depending on the predator species, developmental stage of the predator or prey, prey size etc. [60–62]. In line with our prediction, the presence of C+ sawflies was beneficial for C− sawflies, with a smaller proportion of C− sawflies consumed in group-composition treatments that had C+ individuals present compared to groups of C− sawflies only. This suggests that the presence of chemically defended sawflies can lead to protection from predation for conspecifics. Such protection may result from the C− individuals acquiring clerodanoids, or temporary learned avoidance of C− by mantids after encountering C+ sawflies. Our chemical analysis showed that most, but not all, sawflies had acquired detectable amounts of clerodanoids, suggesting that both of these mechanisms could play a part in the protection from predation for C− conspecifics. There was no significant change in the expected direction in number of consumed C− sawflies across the gradient of the C+ and C− mixed group-composition treatments. This may have been due to the low total number of sawflies used (six), and hence only small differences between the mixed treatments. The domestic chick, *Gallus gallus domesticus*, rejected mimics (palatable prey items) less frequently when the relative abundance of these mimics compared to the models (unpalatable prey items) increased, but birds only discriminated between the models and mimics when the frequency of mimics was above 25% [63]. Moreover, density-dependence of predation can also be influenced by other factors such as predator behaviour, e.g. learning, forgetting and memory [64], and the energetic and informational state of the predator, leading to state-dependent decision-making [65].

In natural populations of *A. rosae*, intraspecific variation in clerodanoid uptake could be expected if the distribution of pharmacophagy-suitable plants is patchy, if there is intraspecific variation in the clerodanoid concentrations available from the plants, or if there are associated costs of clerodanoid uptake. Indeed, *A. rosae* individuals that were exposed to clerodanoids had a shorter lifespan than control individuals [39]. This suggests that there might be costs to clerodanoid uptake, although clerodanoid access did not immediately cause high mortality during our observed period in experiment 2 (figure 2*b*). Similar costs of chemical defense have been revealed in swallowtail butterflies, which showed reduced larval survivorship [24] or a reduction in adult fat content [66] when sequestering alkaloids. Our study demonstrates that even if individuals do not take up clerodanoids, they could still benefit from conspecifics that do. This may lead to the emergence of cheaters that do not pay the cost of chemical defense but enjoy its benefits [67]. Future studies should examine variation in clerodanoid contents in natural populations of *A. rosae*.

## Data Availability

All data of this article are available online at https://doi.org/10.5061/dryad.mpg4f4r20 [68]. Electronic supplementary material is available online [69].
